# Changes of diaphragmatic excursion and lung compliance during major laparoscopic pelvic surgery: A prospective observational study

**DOI:** 10.1371/journal.pone.0207841

**Published:** 2018-11-29

**Authors:** Kyungmi Kim, Dong-Min Jang, Jong-Yeon Park, Hwanhee Yoo, Hong Soon Kim, Woo-Jong Choi

**Affiliations:** 1 Department of Anesthesiology and Pain Medicine, Gil Medical Center, Gachon University College of Medicine, Incheon, Republic of Korea; 2 Department of Anesthesiology and Pain Medicine, Asan Medical Center, University of Ulsan College of Medicine, Seoul, Republic of Korea; ASST Melegnano-Martesana, ITALY

## Abstract

Major laparoscopic pelvic surgery requires steep Trendelenburg position with pneumoperitoneum for a long time. We investigated the effect of Trendelenburg position with pneumoperitoneum on diaphragmatic excursion and lung compliance during major laparoscopic pelvic surgery using M-mode sonography. Twenty patients undergoing elective pelviscopic radical hysterectomy were included in this study. Diaphragmatic excursion was measured at the following time points; after sedation, after intubation, 90 minutes after Trendelenburg position with pneumoperitoneum, and after operation with recovery of muscle relaxation. And lung compliance was measured using anesthetic machine under general anesthesia; after the intubation, 90 minutes after Trendelenburg position with pneumoperitoneum and after operation with recovery of muscle relaxation. In order to detect postoperative pulmonary complication, postoperative chest radiography was checked. Static lung compliance, dynamic lung compliance and diaphragmatic excursion were decreased during operation (*P* < 0.001, respectively). At the end of the operation with recovery of muscle relaxation, reduced diaphragmatic movement was not recovered as its excursion after sedation (*P* < 0.001). In conclusion, lung compliance was decreased following transiently decreased diaphragmatic excursion during major laparoscopic pelvic surgery.

## Introduction

In recent years, laparoscopic surgery has been preferred to open techniques because it results in less incisional pain, fewer pulmonary complications, and shorter hospital stays [1, 2]. However, pneumoperitoneum decreases pulmonary compliance due to cephalad displacement of the diaphragm [3]. Cephalad displacement of the diaphragm can incur intraoperative lung volume changes, consequently leading to the possibility of atelectasis formation [4]. Diaphragmatic movement may also decrease after maintaining the steep Trendelenburg position with pneumoperitoneum for a long time. Previous studies reported that impaired diaphragmatic function after abdominal surgery is a determining factor in the pathogenesis of postoperative pulmonary dysfunction [5, 6]. However, the extent to which diaphragmatic excursion decreases in major laparoscopic pelvic surgery has not been reported yet. Therefore, the impact of the Trendelenburg position with pneumoperitoneum on diaphragmatic excursion during major laparoscopic pelvic surgery needs to be investigated. M-mode sonography can be used to quantitatively assess diaphragmatic excursion as a reflection of diaphragmatic function [7]. M-mode sonography is the easiest method for performing this assessment, and it has high intra- and interobserver agreement [7, 8]. Thus, we investigate the compliance of lung following change of diaphragmatic excursion during major laparoscopic pelvic surgery with steep Trendelenburg position and pneumoperitoneum by using M-mode sonography.

## Materials and methods

### Subjects

After approval by the institutional Ethics Committee (Asan Medical Center Institutional Review Board 2015–0494, Seoul, Korea) and registration in the Korean Clinical Trials Registry (KCT0001710), written informed consent was obtained from all patients. Twenty adult patients (ASA physical status I-II) who underwent elective pelviscopic radical hysterectomy were prospectively included in this study. Patients with chronic obstructive pulmonary disease or respiratory dysfunction were excluded, and patients who underwent conversion to laparotomy or who had a poor echo window dropped out of the study.

### Sample size and clinical data

Based on a pilot study, the mean difference of diaphragmatic excursion before and after the intubation was 4.7 ± 4.5 mm. In a sample size calculation where α was 0.05, β was fixed at 0.1, and a 10% dropout rate was assumed, it was determined that this study required 20 patients. The demographic variables examined included patient age, body mass index, and history of hypertension, diabetes, and other systemic diseases. Intraoperative data were defined as operative time, arterial partial pressure of oxygen, arterial partial pressure of carbon dioxide, end-tidal carbon dioxide pressure. Pulmonary parameters such as peak inspiratory pressure and lung compliance were measured using an anesthesia machine attached ventilator (Primus, Dragger, Lubeck, Germany). Chest X-ray (CXR) within 15 postoperative days was checked to estimate postoperative pulmonary complication. All clinical data for the 20 patients who underwent elective laparoscopic radical hysterectomy were obtained from the electronic medical records system of our institution.

### Anesthesia and mechanical ventilation

General anesthesia was maintained during surgery according to the standardized management protocol of our institution as follows. Anesthesia was induced using 4–5 mg/kg pentothal sodium, 0.6 mg/kg rocuronium, and 3.0–6.0 ng/ml effective site concentration remifentanil via target-controlled infusion using the Orchestra Base Primea target-controlled infusion system (Minto pharmacokinetic model [9], Fresinius Kabi, Bad Homburg, Germany). Anesthesia was maintained with a 1.0 minimum alveolar concentrate of desflurane, 2.0 ng/ml effective site concentration remifentanil, and continuous infusion of 0.3 mg/kg/h rocuronium. The dose of 2.0 mg/kg sugammadex was used to reverse muscle relaxation when patients showed the second twitch in response to train-of-four (TOF) stimulation. Standard monitoring included continuous electrocardiograph, heart rate, peripheral oxygen saturation, end-tidal carbon dioxide concentration, and systemic arterial pressure. During surgery, volume-controlled ventilation with 50% of fraction of inspired oxygen was performed with tidal volume set at 8 mL/kg of ideal body weight without positive end expiratory pressure and a respiratory rate of 12 times/min. The I: E ratio was 1: 2 and the plateau time was 10%.

### Measurement of diaphragmatic excursion

**Fig 1:**
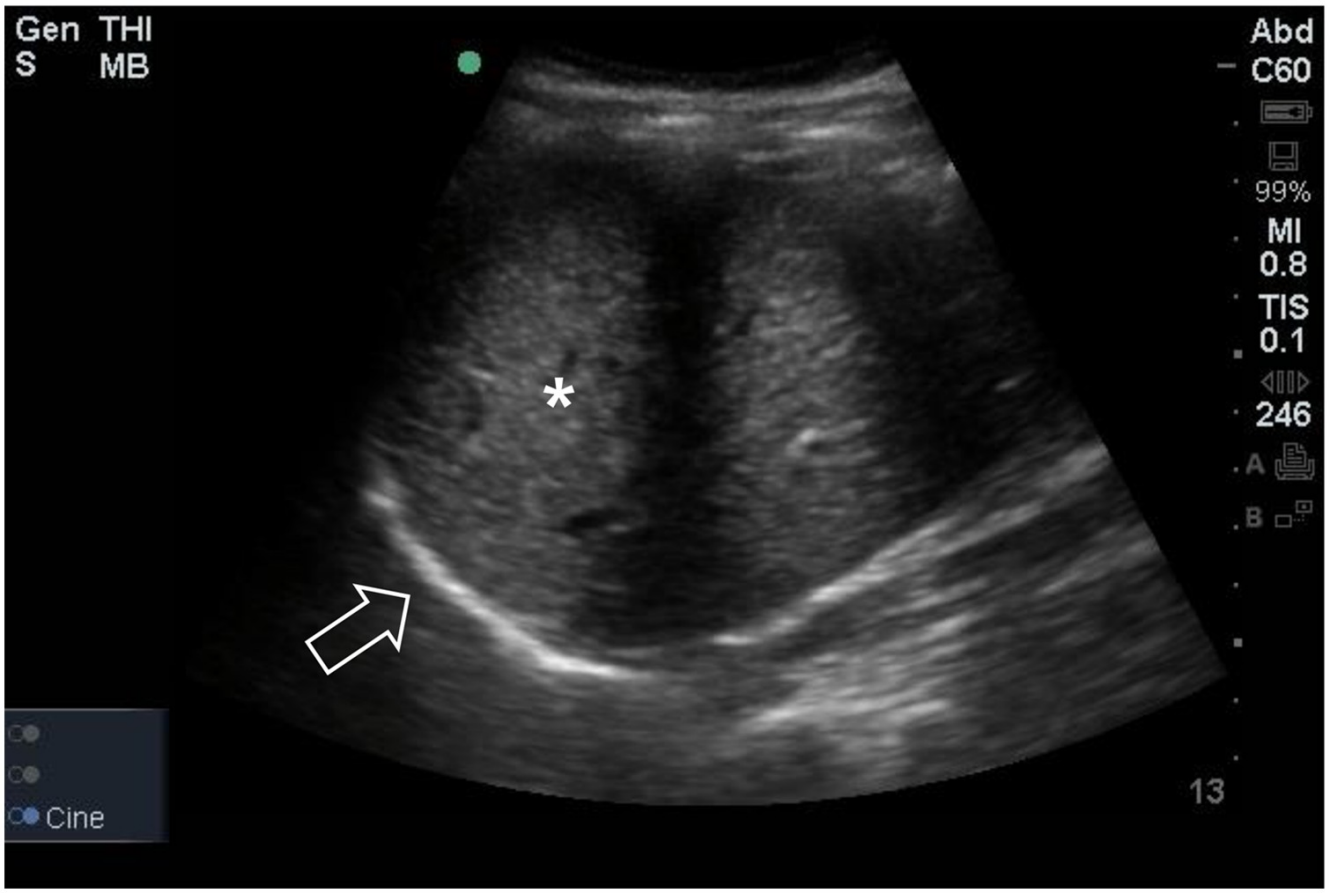
Diaphragmatic excursion as measured using 2D mode sonography. With the probe fixed on the right midaxillary line at approximately the sixth to ninth intercostal spaces, the ultrasound beam was directed to the hemidiaphragmatic domes at an angle of about 70 degrees. The diaphragm (white arrow) is located cephalad to the liver <asterisk>.

**Fig 2:**
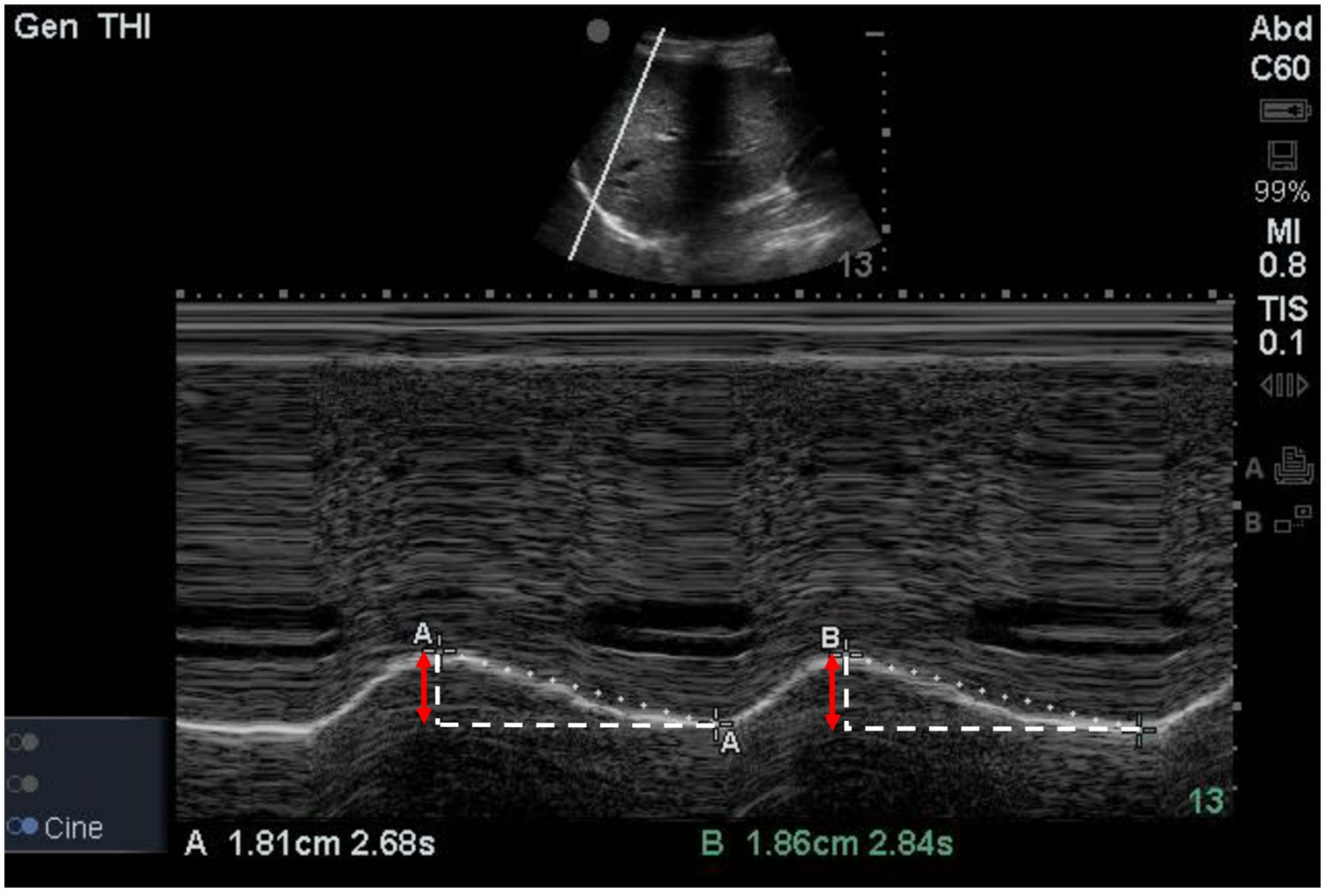
M-mode sonography of diaphragmatic excursion. The amplitude of excursion (red arrow) was measured on the vertical axis of the tracing from the baseline to the point of maximum height of inspiration.

An Edge II ultrasound machine (SonoSite, Inc, Bothell, WA) was used to examine the diaphragm. Diaphragmatic excursion was measured in duplicate by a single well-trained expert (K. K.) with a 5–2 MHz convex transducer to evaluate the impact of muscle relaxation and Trendelenburg position with pneumoperitoneum on diaphragmatic movement. Diaphragmatic excursions were measured after sedation (T0, bispectral index < 60, TOF ratio > 0.9), after the intubation (T1, bispectral index < 60, TOF ratio = 0), 90 minutes after Trendelenburg position with pneumoperitoneum (T2), and after operation with recovery of muscle relaxation under mechanical ventilation (T3, bispectral index < 60, TOF ratio > 0.9). To avoid making an error such as different tidal ventilation at each measurement, T0 was measured while mechanical mask ventilation to maintain the tidal volume as 8 mL/kg of ideal body weight with a respiratory rate of 12 times/min and the other measurements were taken under mechanical ventilation whose tidal volume was set 8 mL/kg of ideal body weight without positive end expiratory pressure. With the probe fixed on the right midaxillary line at approximately between the sixth to ninth intercostal spaces, the ultrasound beam was directed to the hemidiaphragmatic domes at an angle of about 70 degrees [10, 11]. The liver was used as the marker of window for the right hemidiaphragm (Fig 1). M-mode sonography was used to assess the diaphragmatic excursion. The amplitude of excursion was measured on the vertical axis of the tracing from the baseline to the point of maximum height of inspiration on the graph (Fig 2) [7]. Measurements were recorded in duplicate and averaged for each time period.

## Statistical analysis

Continuous variables are expressed as mean ± standard deviation, and categorical variables are summarized as number and percentage. Continuous variables were analyzed using the Student t-test. Repeated measures ANOVA followed by Bonferroni correction was used to analyze serial change of pulmonary variables. Data manipulation and statistical analyses were performed using SPSS 21.0 (SPSS Inc., Chicago, IL).

## Results

Twenty patients were enrolled in the present study, but two patients dropped out: one patient who underwent conversion to laparotomy and another patient with a poor echo window due to subcutaneous emphysema. Demographic and perioperative characteristics of the study patients are reported in Table 1.

**Table 1:** Demographic and perioperative characteristics of the study patients.

	Mean or Numbers (n = 18)
**Age (years)**	49.4 ± 9.0
**Body mass index (kg/m**^**2**^**)**	22.8 ± 3.5
**Hypertension**	2 (11.1%)
**Diabetes mellitus**	0 (0%)
**Other systemic disease**	3 (16.7%)
**Hospital stay (days)**	10.2 ± 7.9
**Abnormal CXR finding within 15 postoperative days**	7 (38. 9%)
**Intraoperative Data**	
**Operative time (min)**	226.1 ± 68. 0
**Admitted crystalloid (mL)**	2352.8 ± 789.2

Data are expressed as mean ± standard deviation or number (percentage).

CXR = chest X-ray; abnormal CXR finding = atelectasis or effusion

Mean length of hospital stay was 10.2 ± 7.9 days. Postoperative abnormal CXR findings (atelectasis or pleural effusion) were presented in 7 patients, and there were no complaints of respiratory discomfort or pneumonia. Table 2 shows changes of intraoperative pulmonary values.

**Table 2:** Pulmonary variables during the operation.

	T1	T2	T3	*P* value
**PaO**_**2**_ **on FiO**_**2**_ **0.5 (mmHg)**	240.2 ± 52.4	204.6 ± 40.2	210.6 ± 39.0	0.011
**PaCO**_**2**_ **(mmHg)**	35.8 ± 4.1	40.7 ± 4.7	39.3 ± 5.5	0.030
**EtCO**_**2**_ **(mmHg)**	30.6 ± 2.7	33.1 ± 4.2	32.9 ± 2.9	0.026
**PIP (mmH**_**2**_**O)**	12.8 ± 2.0	25.6 ± 2.8	15.9 ± 2.1	< 0.001
**Static Compliance (ml/mmH**_**2**_**O)**	35.2 ± 6.1	18.1 ± 3.3	29.0 ± 4.7	< 0.001
**Dynamic Compliance (ml/mmH**_**2**_**O)**	33.9 ± 5.0	17.2 ± 2.2	27.5 ± 3.2	< 0.001

Data are expressed as mean ± standard deviation

T1 = after the intubation (bispectral index < 60, train-of-four (TOF) ratio = 0); T2 = 90 minutes after Trendelenburg position with pneumoperitoneum; T3 = after operation with recovery of muscle relaxation under mechanical ventilation (bispectral index < 60, TOF ratio > 0.9); PaO_2_ = arterial partial pressure of oxygen; FiO_2_ = fraction of inspired oxygen; PaCO_2_ = arterial partial pressure of carbon dioxide; EtCO_2_ = end-tidal carbon dioxide concentration; PIP = peak inspiratory pressure measured by an anesthesia machine (Primus, Dragger, Lubeck, Germany); Compliance = pulmonary compliance measured by an anesthesia machine (Primus, Dragger, Lubeck, Germany)

**Fig 3:**
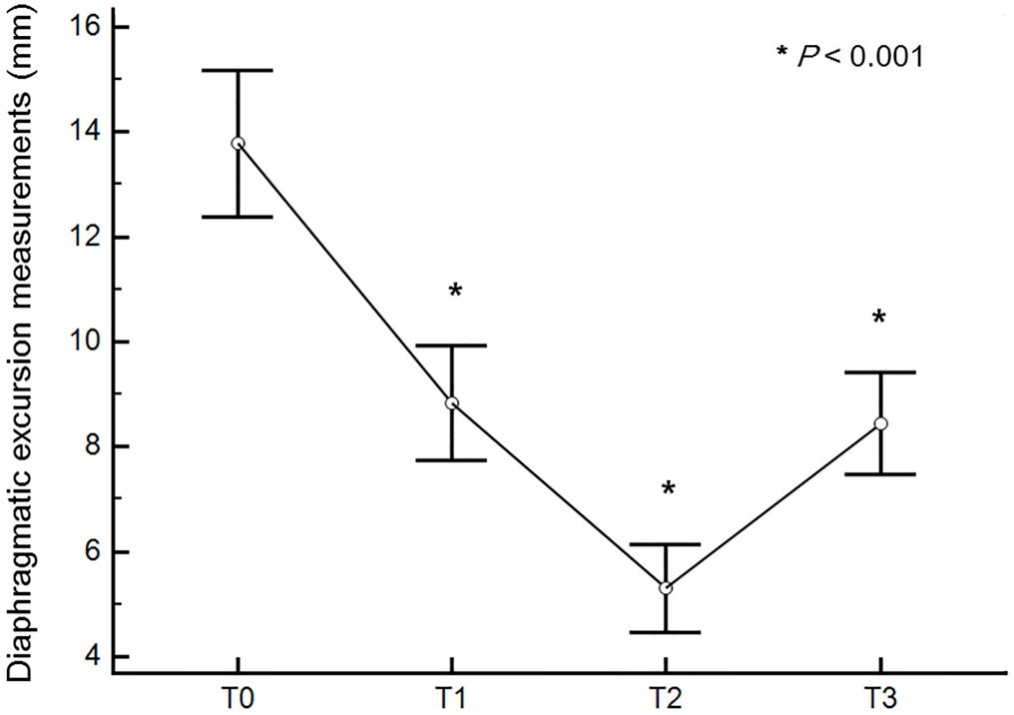
Diaphragmatic excursions at each surgical time point. Diaphragmatic movement was decreased gradually during operation. Diaphragmatic excursion had the largest drop 90 minutes after Trendelenburg position with pneumoperitoneum (T2) compared to after sedation (T0, *P* < 0.001). T0 = after sedation (bispectral index < 60, train-of-four (TOF) ratio > 0.9); T1 = after the intubation (bispectral index < 60, TOF ratio = 0); T2 = 90 minutes after Trendelenburg position with pneumoperitoneum; T3 = after operation with recovery of muscle relaxation under mechanical ventilation (bispectral index < 60, TOF ratio > 0.9).

PaO_2_ was decreased transiently during pneumoperitoneum with steep Trendelenburg position and recovered at end of surgery (*P* = 0.011). Peak inspiratory pressure was significantly increased during operation (*P* < 0.001). There was significant decrease of pulmonary compliance during operation (*P* < 0.001). Diaphragmatic excursion was, also, decreased gradually during operation (*P* < 0.001, Fig 3).

## Discussion

Laparoscopic surgery has resulted in shorter hospital stay, less pulmonary complication, less postoperative pain than open surgery [1, 2]. However, this study revealed that lung compliance was also decreased following transiently decreased diaphragmatic excursion during major laparoscopic pelvic surgery with steep Trendelenburg position and pneumoperitoneum. Thus, diaphragmatic excursion may be one of the critical factors to maintain pulmonary function during major laparoscopic pelvic surgery.

It is well known that general anesthesia and abdominal surgery leads to decreased respiratory function [4, 12]. Magnusson and Spahn concluded compression atelectasis occurred during general anesthesia and was caused by chest geometry and diaphragm position and motion [13]. Pneumoperitoneum also affected cephalic displacement of diaphragm and decreased functional residual capacity [14]. In agreement with previous studies, our study revealed that major laparoscopic pelvic surgery not only decreased lung volume due to cephalic displacement of the diaphragm, but also decreased diaphragmatic movement. Our results showed diaphragmatic excursion was decreased gradually during operation (*P* < 0.001). Although we observed diaphragmatic excursion only during operation, reduced diaphragmatic movement at the end of operation was not recovered as its excursion after sedation. Kim et al. found that there was a significant decrease in postoperative diaphragmatic inspiratory amplitude values compared to preoperative values [15]. It may contribute to limited recovery of diaphragmatic movement after surgery. Likewise, several reports suggested that diaphragmatic impairment after laparoscopic surgery is caused by gas insufflation in the abdominal cavity, which might also be responsible for the increased resistance and reduced diaphragmatic excursion, leading to reduced lung volume [16]. In contrast with Erice and his colleagues, who reported that diaphragmatic function was not changed in patients who underwent minor low abdominal laparoscopic surgery [17], our data presented diaphragmatic movement was decreased after intubation and gradually reduced during laparoscopic pelvic surgery with Trendelenburg position and pneumoperitoneum. Our study enrolled the patients undergoing elective pelviscopic radical hysterectomy for a duration of at least 2 hours and general anesthesia lead to decrease diaphragmatic excursion and prolonged Trendelenburg position with pneumoperitoneum further restricted the reduced movement of diaphragm. Therefore, major laparoscopic pelvic surgery might contribute to transient reduction of diaphragmatic excursion.

In this study, lung compliance was also decreased following transiently decreased diaphragmatic excursion during major laparoscopic pelvic surgery even if muscle relaxation had fully recovered. Postoperative atelectasis or pleural effusion, which was defined the evidence of decreased lung volume, was shown in seven patients (38.9%). It might be a result of reduced diaphragmatic movements during laparoscopic surgery rather than thoracic rib cage movements. Consistent with the findings of our present study, Cakmakkaya et al. demonstrated that lung compliance after laparoscopic surgery was not fully restored to baseline level [18]. They found a simple alveolar recruit maneuver was helpful to improve lung compliance after laparoscopic surgery. Spadaro and his colleagues, also, reported aggressive positive end-expiratory pressure was required to improve pulmonary compliance during laparoscopic surgery [19]. We only observed diaphragmatic excursions during operation nevertheless applying recruitment maneuver or positive end-expiratory pressure might affect diaphragmatic excursion and respiratory mechanics. It needs additional studies to improve transient reduction of diaphragmatic excursion and lung compliance during laparoscopic surgery.

Our study had some limitations. First, there was some bias in study design. This study was a single-centre observational study that might have possible selection bias. The population of this study consisted of previous healthy patients (ASA physical status I, II), and all patients were Asian, primarily Korean. Despite it was well known that effects of anesthesia and paralysis on diaphragmatic mechanics [20], we did not assess the single effect of general anesthesia on diaphragmatic movement nor did we measure diaphragmatic excursion at different condition such as decreased pneumoperitoneum pressure or low degree muscle relaxation. Second, we could not evaluate lung compliance such as remifentanil induced chest wall rigidity. According to Ri and his colleagues, however, chest wall rigidity was occurred by the effect site concentration of remifentanil 4.0 ng/mL in 45% of Korean patients [21]. Remifentanil was maintained 2.0 ng/ml effective site concentration during operation and we assessed diaphragmatic movement when patients were sedated using only pentothal sodium (T0) and fully relaxed because of muscle relaxant (T1—T2, TOF ratio = 0), and after operation with stopping remifentanil infusion (T3). Finally, we measured diaphragmatic excursion only in the intraoperative period, so that this study could not confirm postoperative pulmonary dysfunction even though our study suggested that Trendelenburg position with pneumoperitoneum affected diaphragmatic excursion and lung compliance during laparoscopic surgery. We are continually working on assessment of the impact of Trendelenburg position with pneumoperitoneum on diaphragmatic excursion and lung compliance during laparoscopic surgery. It might be helpful to substantiate the maximal tolerable value for special condition such as patients with pulmonary disease or obesity.

## Conclusions

In conclusion, diaphragmatic excursion and lung compliance were decreased during major laparoscopic pelvic surgery with steep Trendelenburg position and pneumoperitoneum. The reduced lung compliance may impact on postoperative pulmonary function in patients with major laparoscopic pelvic surgery. Further study is needed to evaluate clinical implication.

## Supporting information

S1 Dataset (XLSX)

## Abbreviations

CXR: chest X-ray
TOF: train-of-four
